# A GH51 α-l-arabinofuranosidase from *Talaromyces leycettanus* strain JCM12802 that selectively drives synergistic lignocellulose hydrolysis

**DOI:** 10.1186/s12934-019-1192-z

**Published:** 2019-08-19

**Authors:** Tao Tu, Xiaoli Li, Kun Meng, Yingguo Bai, Yuan Wang, Zhenxing Wang, Bin Yao, Huiying Luo

**Affiliations:** Key Laboratory for Feed Biotechnology of the Ministry of Agriculture, Feed Research Institute, Chinese Academy of Agricultural Sciences, No. 12 Zhongguancun South Street, Beijing, 100081 People’s Republic of China

**Keywords:** *Talaromyces leycettanus* JCM12802, α-l-Arabinofuranosidase, Saccharification, Oligosaccharides

## Abstract

**Background:**

The development of sustainable technologies for plant cell wall degradation greatly depends on enzymes with hydrolytic activities against carbohydrates. The waste by-products of agricultural cereals are important biomass sources because they contain large amounts of saccharides. Achieving efficient debranching and depolymerization are two important objectives for increasing the utilization of such renewable bioresources. GH51 α-l-arabinofuranosidases are important in biomass pretreatment because they act synergistically with other enzymes during hemicellulose hydrolysis.

**Results:**

A GH51 α-l-arabinofuranosidase from *Talaromyces leycettanus* JCM12802 was heterologously expressed in *Pichia pastoris* GS115 and characterized. The recombinant α-l-arabinofuranosidase, *Tl*Abf51, showed an optimum temperature and pH of 55–60 °C and 3.5–4.0, respectively, and remained stable at 50 °C and pH 3.0–9.0. *Tl*Abf51 showed a higher catalytic efficiency (5712 mM^−1^ s^−1^) than most fungal α-l-arabinofuranosidases towards the substrate 4-nitrophenyl-α-l-arabinofuranoside. Moreover, *Tl*Abf51 preferentially removed 1,2- or 1,3-linked arabinofuranose residues from arabinoxylan and acted synergistically with the bifunctional xylanase/cellulase *Tc*Xyn10A at an activity ratio of 5:1. The highest yields of arabinose and xylooligosaccharides were obtained when *Tl*Abf51 was added after *Tc*Xyn10A or when both enzymes were added simultaneously. High-performance anion-exchange chromatography analyses showed that (i) arabinose and xylooligosaccharides with low degrees of polymerization (DP1–DP5) and (ii) arabinose and xylooligosaccharides (DP1–DP3) were the major hydrolysates obtained during the hydrolysis of sodium hydroxide-pretreated cornstalk and corn bran, respectively.

**Conclusions:**

In contrast to other fungal GH51 α-l-arabinofuranosidases, recombinant *Tl*Abf51 showed excellent stability over a broad pH range and high catalytic efficiency. Moreover, *Tl*Abf51 acted synergistically with another hemicellulase to digest arabino-polysaccharides. These favorable enzymatic properties make *Tl*Abf51 attractive for biomass pretreatment and biofuel production.

**Electronic supplementary material:**

The online version of this article (10.1186/s12934-019-1192-z) contains supplementary material, which is available to authorized users.

## Background

The large amounts of by-products produced during the machining of agricultural cereals, such as straw, stover, and husks, are important resources for biofuel production [1]. Lignocellulosic biomass, as the most abundant renewable bioresource, is derived from plant cell walls and is mainly comprised of cellulose, hemicellulose, and lignin, among which cellulose and hemicellulose are the first and second most abundant polysaccharides on Earth [2, 3]. Because hemicellulose is concatenated with cellulose via hydrogen bonds and is chemically cross-linked with lignin, it is naturally resistant to digestion by cellulases. Thus, hemicellulose degradation can help cellulases access cellulose, resulting in more efficient cellulose utilization [4, 5]. Therefore, a more efficient means of enzymatic hemicellulose depolymerization in the biofuel and biorefinery industries is desired.

The main component of hemicellulose is xylan, which is composed of covalently β-1,4-linked d-xylose residues that can be attached by substituents at different side chains such as l-arabinose, d-glucuronic acid, 4-*O*-methyl-d-glucuronic acid, ferulic acid, *p*-coumaric acid, and acetyl groups [6]. Thus, thorough degradation or modification of xylan requires the combined activities of several different enzymes, including the core enzymes endo-β-1,4-d-xylanase (EC 3.2.1.8) and β-1,4-d-xylosidase (EC 3.2.1.37) along with other accessory enzymes, such as α-l-arabinofuranosidase (Abf, EC 3.2.1.55). However, complex substrates with branched side chains are not easily degraded. Thus, an accessory enzyme, such as Abf, is valuable for industrial applications such as plant residue biotransformation, food processing, and pulp bleaching.

Abfs are normally found in six glycoside hydrolase (GH) families: GH2, GH3, GH43, GH51, GH54, and GH62. These families are divided based on sequence similarity and differentiated based on their modes of action against substrates with different linkages [7]. Generally, Abf members catalyze the hydrolysis of arabinose from the non-reducing ends of different arabinose-containing polysaccharides and oligosaccharides (α-1,2-, α-1,3-, and α-1,5-; [8]). Based on their substrate specificity, Abfs are grouped into three types (A, B, and C). Type A Abfs preferentially act on arabinooligosaccharides, while type B Abfs preferentially act on both polysaccharides and arabinooligosaccharides. Both type A and B Abfs show activity towards *p*-nitrophenyl-α-l-arabinofuranoside. In contrast, type C Abfs specifically degrade arabinosidic linkages within arabinoxylans [9]. Fungal GH51 Abfs from *Aspergillus awamori* IFO4033 [10], *Aspergillus nidulans* FGSC A4 [11], *Aspergillus niger* CBS 513.88 [12], *Chrysosporium lucknowense* C1 [13], and *Penicillium chrysogenum* 31B [14] have been characterized. These Abfs are active against arabinoxylans and arabino-containing saccharides and release arabinose, although they show much higher activity towards branched arabinan compared to debranched arabinan [15]. These applaudable enzymatic characteristics make GH51 Abfs interesting for synergistic use with other hemicellulases to completely degrade hemicelluloses.

*Talaromyces leycettanus* strain JCM12802 produces high levels of cellulases (such as β-glucanase [16] and β-mannanase [17]), hemicellulases (such as xylanase [18]), and pectinases (such as polygalacturonase [19]). In this study, we identified an Abf gene (*Tlabf51*) of GH51 in *T. leycettanus* JMC12802, which was overexpressed in *Pichia pastoris* GS115 and characterized. Next, we studied the synergistic activities of *Tl*Abf51 and the bifunctional xylanase/cellulase *Tc*Xyn10A (from *Thermoascus crustaceus* JCM12803; [20]) in the hydrolysis of water-soluble wheat arabinoxylan. The synergistic effects on the degradation of sodium hydroxide-pretreated cornstalk and corn bran were also investigated.

## Results

### Gene cloning and sequence analysis

An Abf, designated here as *Tl*Abf51, was isolated from the thermophilic *T. leycettanus* JCM12802. The full-length chromosomal and cDNA sequences of *Tlabf51* (GenBank accession no. MK734377) consisted of 2362 and 1887 base pairs, respectively. Eight introns interrupted the cDNA sequence and the mature protein contained 628 residues with a calculated molecular mass of 67.2 kDa. Seven putative *N*-glycosylation sites and four *O*-glycosylation sites were identified within the deduced *Tl*Abf51 sequence by NetNGlyc Server analysis. The deduced amino acid sequence of *Tl*Abf51 shares the highest identity of 79.9% with the glycoside hydrolase of *Aspergillus ellipticus* CBS 707.79 and 28.1% sequence identity with the known crystal structure of Abf from *Bifidobacterium longum* (2Y2W), demonstrating that *Tlabf51* is a novel Abf gene.

### Expression and purification of recombinant *Tl*Abf51

Recombinant *Tl*Abf51 was expressed in the *P. pastoris* GS115 system and secreted into the culture medium. Significant Abf activities were observed in shake tube cultures against 4-nitrophenyl-α-l-arabinofuranoside. After large-scale cultivation and purification, the electrophoretic homogeneity of recombinant *Tl*Abf51 was determined by sodium dodecyl sulfate-polyacrylamide gel electrophoresis (SDS-PAGE) analysis (Additional file 1). To verify the target protein, liquid chromatography-electrospray ionization tandem mass spectrometry (LC–ESI-MS) was conducted to identify the band. Five peptides corresponding to the sequence of recombinant *Tl*Abf51 and no other peptides were detected (Additional file 2). These results confirmed the purity of the band and the identity of *Tl*Abf51.

### Biochemical characterization

**Fig. 1:**
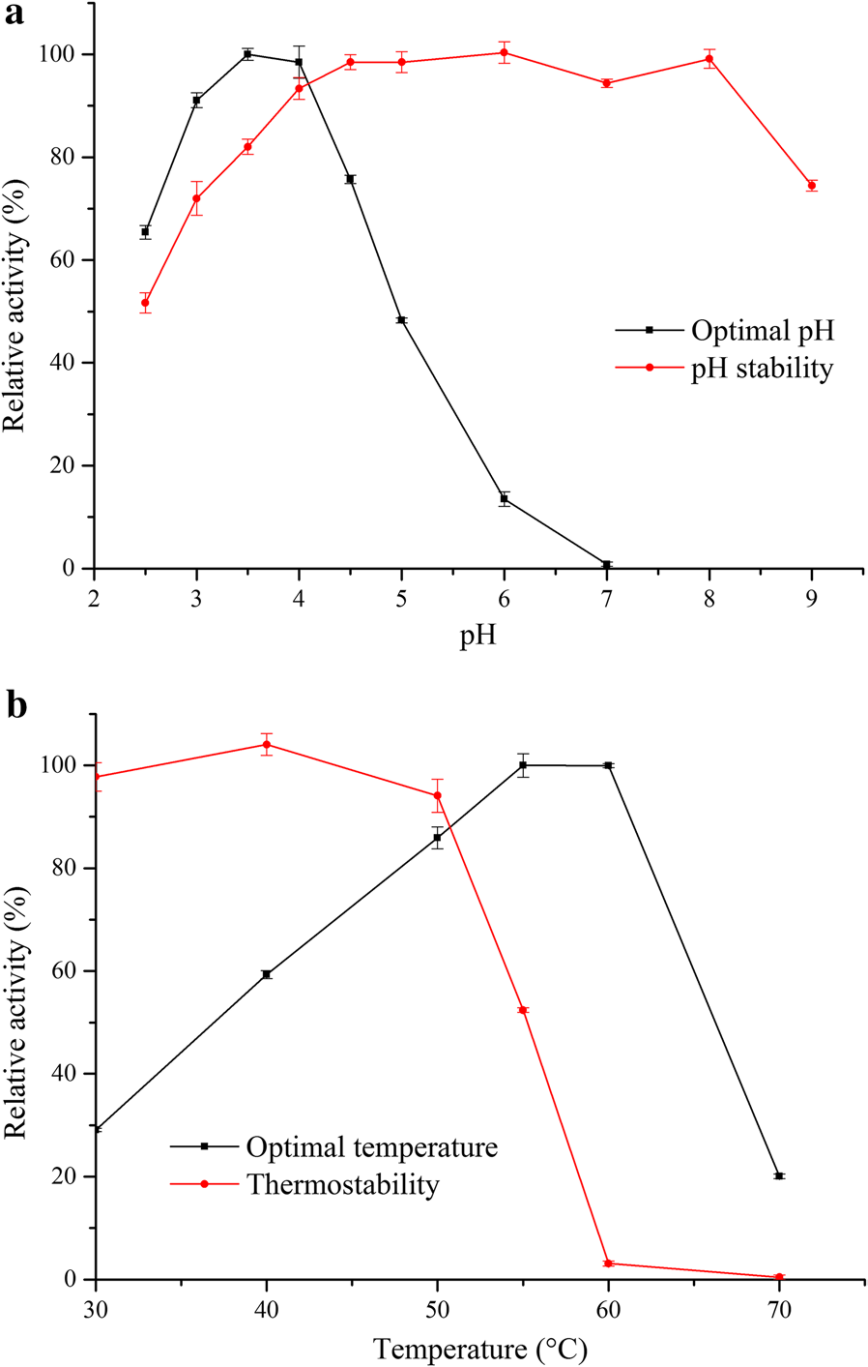
Effects of pH (**a**) and temperature (**b**) on purified recombinant *Tl*Abf51. The activity of *Tl*Abf51 was measured under the indicated conditions and presented as a percentage of the maximum level

For enzyme characterization, 4-nitrophenyl-α-l-arabinofuranoside was used as a substrate. *Tl*Abf51 was optimally active at pH 3.5–4.0 and showed > 40% of its peak activity at pH 2.5–5.0 (Fig. 1a). The enzyme exhibited good stability over a wide pH range, maintaining > 70% of its maximum activity after incubation at pH 3.0–9.0, 37 °C for 1 h (Fig. 1a). The optimal temperature for *Tl*Abf51 activity was 55–60 °C and the enzyme was active over a temperature range of 20–70 °C at pH 3.5 (Fig. 1b). The enzyme showed good thermostability, and > 90% residual activity was retained after 30-min incubation at 50 °C (Fig. 1b). The *T*_50_ value of *Tl*Abf51, i.e., the temperature corresponding to 50% of the peak activity following a 30-min incubation period, was determined to be 55 °C.

### Substrate specificity and kinetic parameters

Several substrates were used to test the substrate specificity of *Tl*Abf51. For 4-nitrophenyl-glycoside substrates, *Tl*Abf51 showed activity only towards 4-nitrophenyl-α-l-arabinofuranoside and 4-nitrophenyl-β-d-xylopyranoside. The relative ratio of activity towards two substrates was 300:1. Under standard conditions, the specific activity of *Tl*Abf51 towards 4-nitrophenyl-α-l-arabinofuranoside was 1068 ± 8.4 U/mg. The *K*_m_, *V*_max_, and *k*_cat_ values of *Tl*Abf51 were determined to be 0.28 ± 0.01 mM, 1428 ± 10.7 μmol min^−1^ mg^−1^, and 1600 s^−1^, respectively. The *k*_cat_/*K*_m_ value was 5712 mM^−1^ s^−1^.

**Fig. 2:**
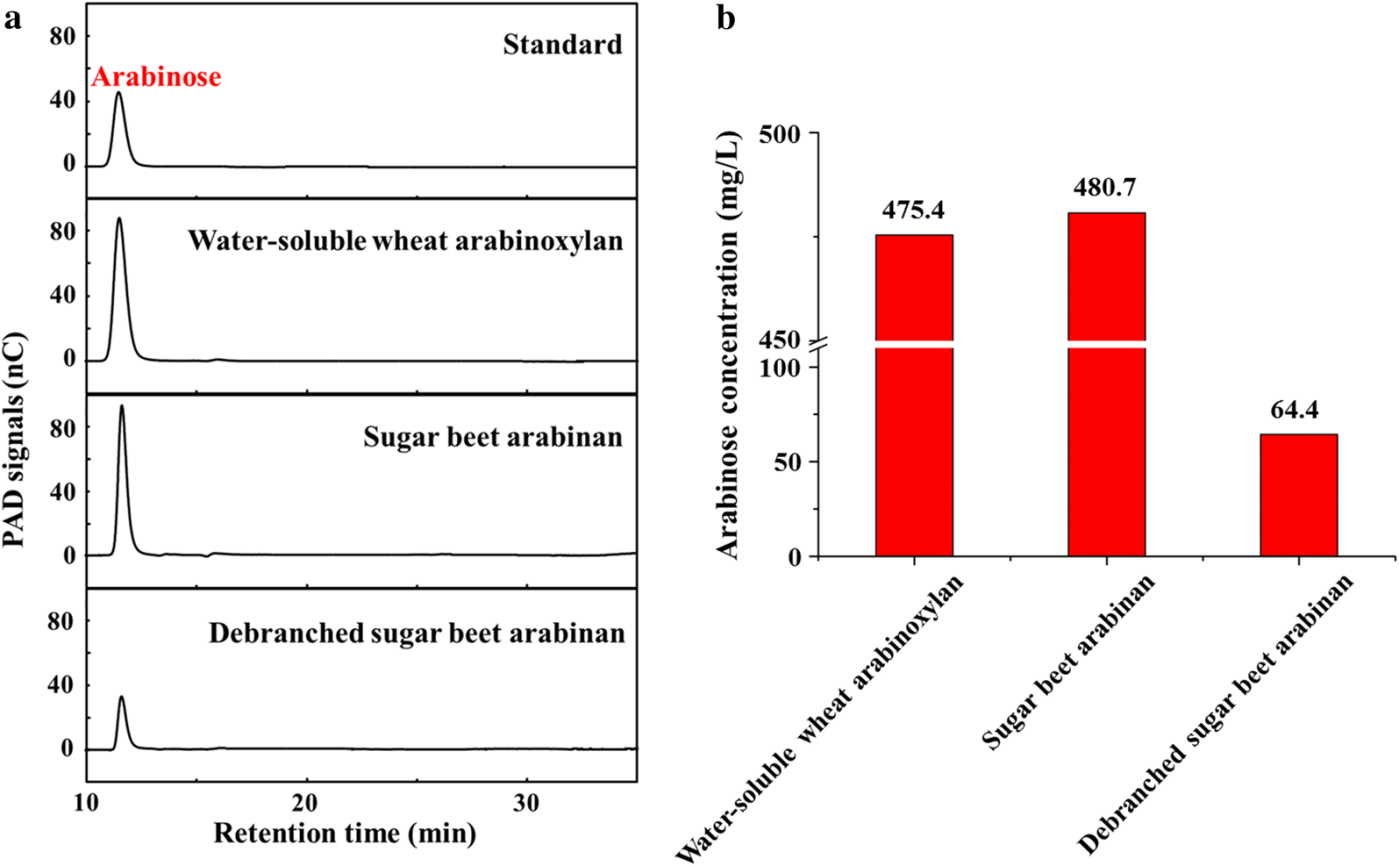
Hydrolytic activity of *Tl*Abf51 on arabinose-based polysaccharide substrates. **a** Water-soluble wheat arabinoxylan, sugar beet arabinan, and debranched sugar beet arabinan were incubated with *Tl*Abf51 at 37 °C for 12 h. The resulting hydrolysates were detected by HPAEC-PAD. **b** The amount of arabinose released by *Tl*Abf51 with each of the three substrates based on the chromatographic peak areas of arabinose shown in **a**. Sugar release was quantified (right panel) using a standard curve for each oligosaccharide

For polysaccharide substrates, *Tl*Abf51 was highly active against water-soluble wheat arabinoxylan (Fig. 2a). After incubation for 12 h, 475.4 mg/L of arabinose was obtained as the final product (Fig. 2b). Moreover, the enzyme exhibited much higher activity towards sugar beet arabinan than against the debranched sugar beet arabinan (releasing 480.7 and 64.4 mg/L arabinose, respectively), indicating that *Tl*Abf51 preferentially acted on 1,2- or 1,3-linked arabinan residues debranched as side chains rather than linear α-1,5-l-arabinan.

### Synergistic effect of *Tl*Abf51 and *Tc*Xyn10A on wheat arabinoxylan degradation

**Fig. 3:**
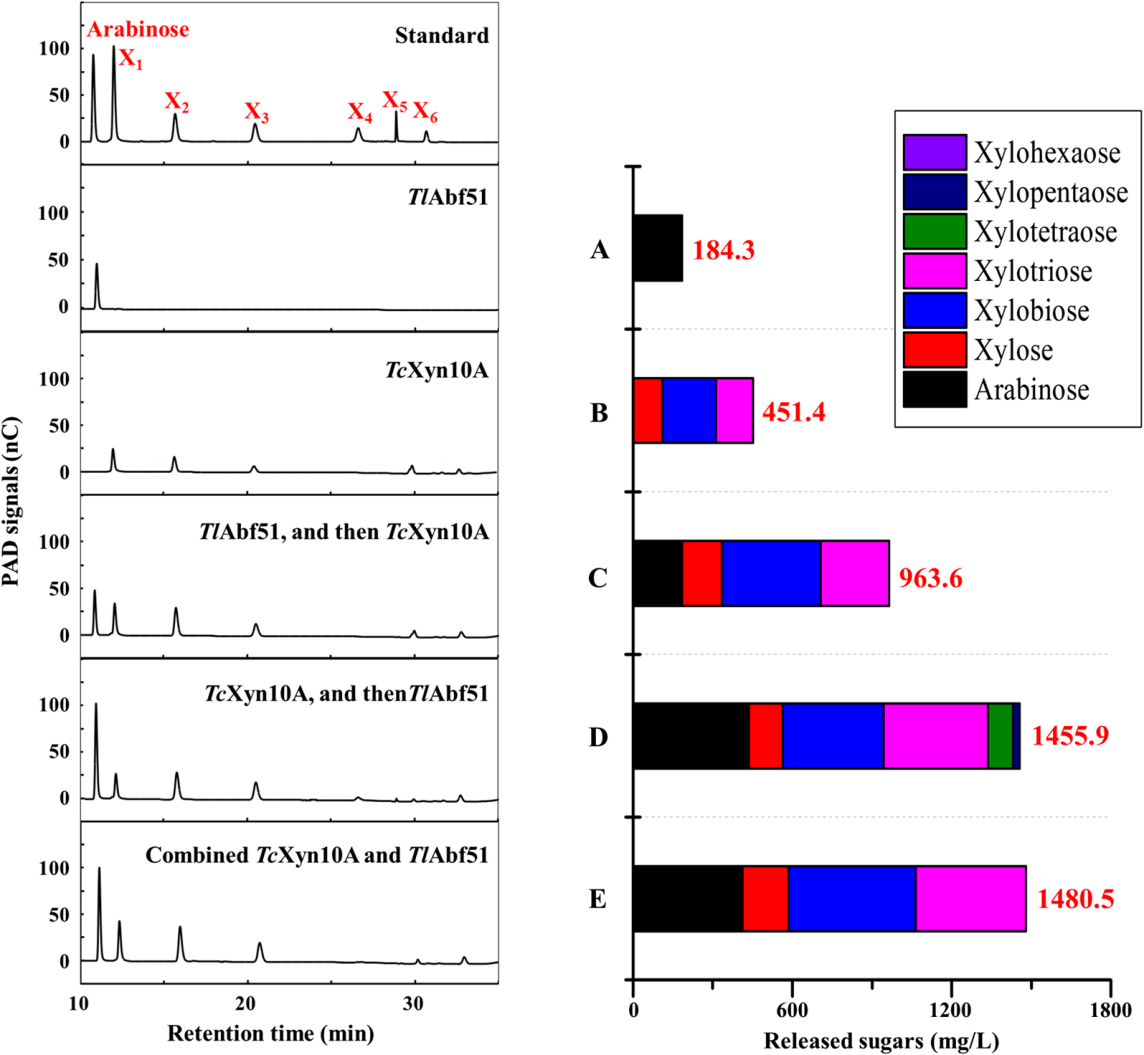
Combined activities of *Tl*Abf51 with the bifunctional xylanase/cellulase *Tc*Xyn10A for water-soluble wheat arabinoxylan degradation. We hydrolyzed 0.5% wheat arabinoxylan using different enzyme combinations, including a reaction with **a** *Tl*Abf51 alone, **b** *Tc*Xyn10A alone, **c** sequential reactions of *Tl*Abf51 and *Tc*Xyn10A for 12 h, respectively, **d** sequential reactions of *Tc*Xyn10A and *Tl*Abf51 for 12 h, respectively, and **e** simultaneous incubation with *Tl*Abf51 and *Tc*Xyn10A for 12 h. The hydrolysates were analyzed by HPAEC-PAD

To improve the efficiency of xylan hydrolysis, the hydrolysis efficiency of water-soluble wheat arabinoxylan by *Tl*Abf51 and bifunctional xylanase/cellulase *Tc*Xyn10A, tested individually or in combination, were analyzed by high-performance anion-exchange chromatography and pulsed amperometric detection (HPAEC-PAD). Compared to the hydrolysis products generated by *Tl*Abf51 or *Tc*Xyn10A alone, all enzyme combinations showed significant synergistic effects on wheat arabinoxylan degradation, producing markedly higher levels of arabinose and xylooligosaccharides. After 12-h incubation of *Tl*Abf51 with 0.5% wheat arabinoxylan, the concentration of arabinose reached 184.3 mg/L (Fig. 3a). When incubated with 0.5 U *Tc*Xyn10A, the levels of xylooligosaccharides (xylose, xylobiose, and xylotriose) reached 451.4 mg/L, although small amounts of arabino-branched xylooligosaccharides were observed (Fig. 3b). Adding *Tl*Abf51 first, followed by *Tc*Xyn10A, resulted in a significant increase in the production of arabinose and xylooligosaccharides (963.6 mg/L) as compared to adding *Tc*Xyn10A alone (Fig. 3c). When *Tc*Xyn10A was added first, followed by *Tl*Abf51, larger amounts of arabinose, xylotetraose, and xylopentaose were released (1455.9 mg/L; Fig. 3d). The greatest synergy was found after simultaneously incubating wheat arabinoxylan with *Tl*Abf51 and *Tc*Xyn10A, with the concentration of released oligosaccharides reaching 1480.5 mg/L (Fig. 3e).

**Fig. 4:**
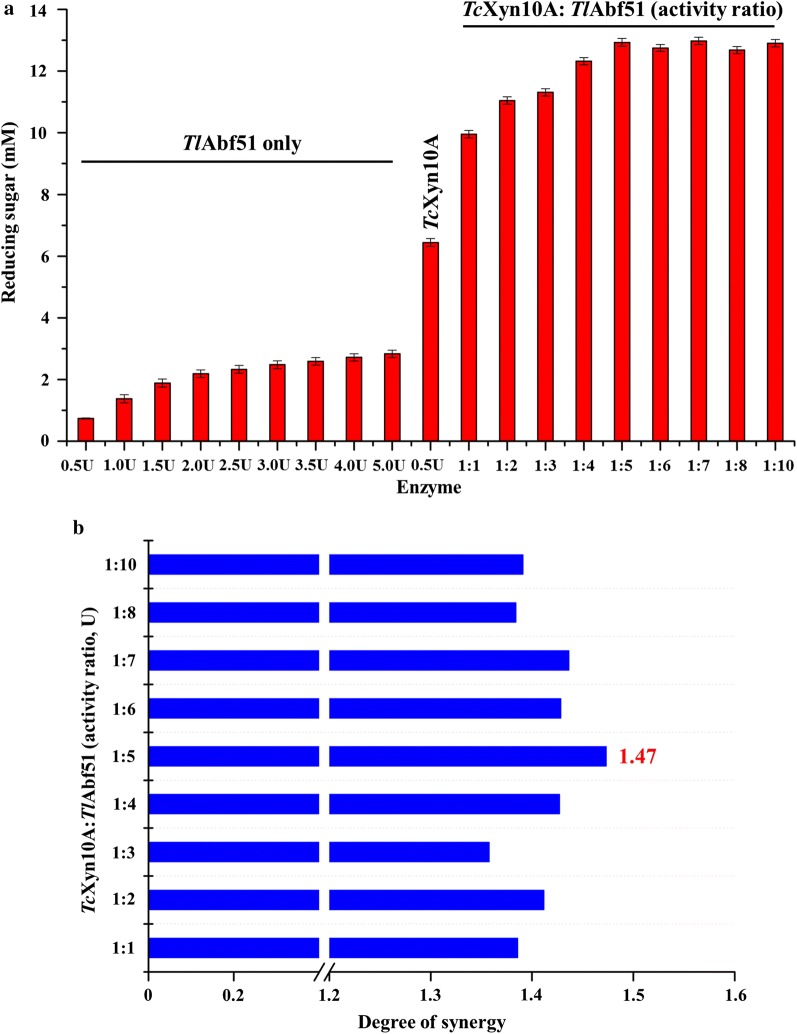
Synergistic effects of *Tl*Abf51 with the bifunctional xylanase/cellulase *Tc*Xyn10A. **a** Reducing sugars obtained by a reaction with *Tl*Abf51 or *Tc*Xyn10A, singly and in combination with different enzyme-activity ratios. **b** Degree of synergy observed after the combined reaction with *Tc*Xyn10A and *Tl*Abf51. The synergic degree was defined as the amounts of saccharides released from the simultaneous activities of both enzymes to the sum of those released by both enzymes individually

To further examine the synergistic effects of *Tl*Abf51 and *Tc*Xyn10A, the released reducing saccharides from water-soluble wheat arabinoxylan by simultaneous incubation with both enzymes were analyzed by the 3,5-dinitrosalicylic acid (DNS) method. *Tl*Abf51 treatment alone liberated reducing sugars from wheat arabinoxylan (Fig. 4a). Higher concentrations of reducing sugars were observed when *Tl*Abf51 and *Tc*Xyn10A were combined at different activity ratios compared to treatment with *Tc*Xyn10A alone. These results suggest that *Tl*Abf51 acted synergistically with *Tc*Xyn10A. The highest degree of synergy was obtained when *Tc*Xyn10A and *Tl*Abf51 were added at an enzyme-activity ratio of 1:5, corresponding to a synergy score of 1.47 (Fig. 4b).

### Hydrolysis of sodium hydroxide-pretreated cornstalk and corn bran with *Tl*Abf51 and *Tc*Xyn10A

**Fig. 5:**
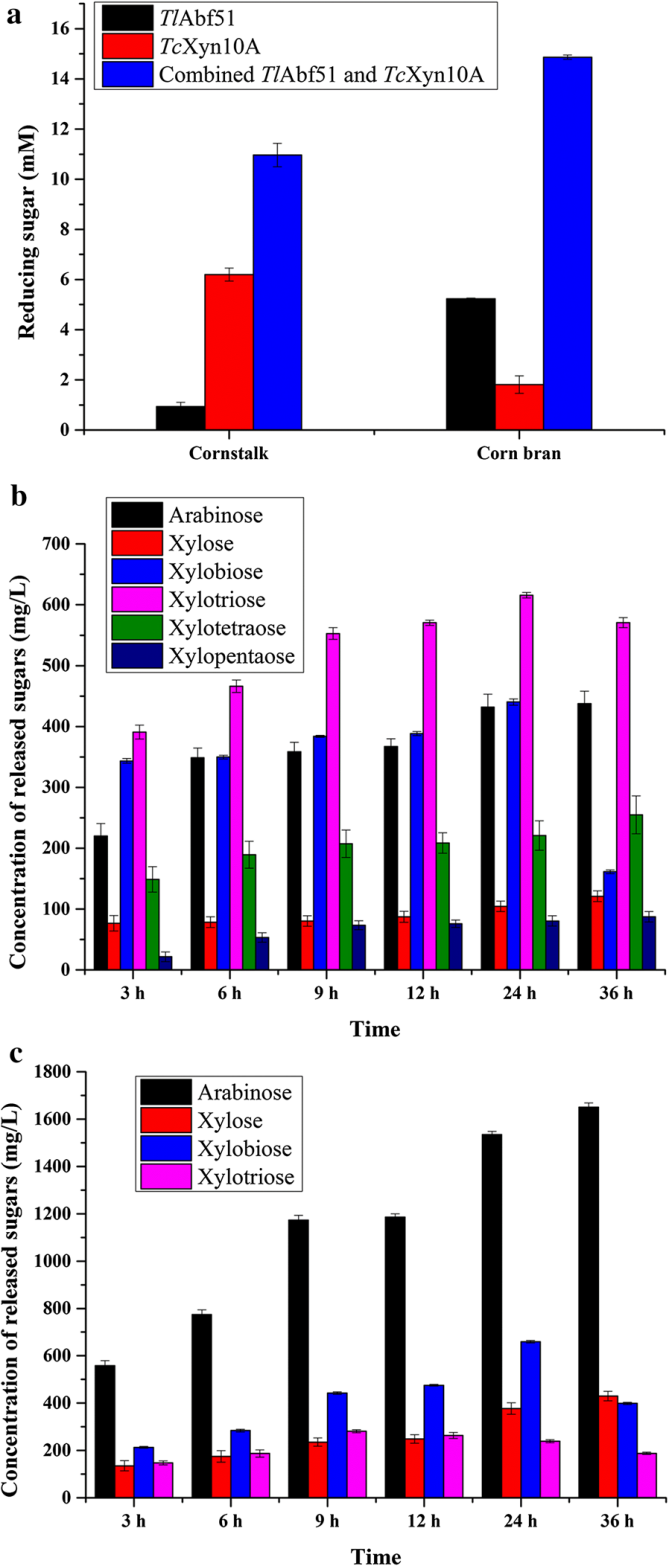
Hydrolysis of sodium hydroxide-pretreated cornstalk and corn bran with *Tl*Abf51 and *Tc*Xyn10A. **a** Reducing sugars obtained by a reaction with *Tl*Abf51 or *Tc*Xyn10A individually or in combination at an enzyme-activity ratio of 1:5 for 3 h. Oligosaccharides were obtained by degradation of hydroxide-pretreated cornstalk (**b**) and corn bran (**c**) in synergistic hydrolysis assays performed for 3, 6, 9, 12, 24 and 36 h

Using sodium hydroxide-pretreated cornstalk and corn bran as respective substrates, *Tl*Abf51 and *Tc*Xyn10A were further tested for their ability to hydrolyze lignocellulose. The reducing sugars in the hydrolysis products obtained after 3-h treatment of sodium hydroxide-pretreated cornstalk and corn bran with *Tl*Abf51 and *Tc*Xyn10A individually or combined at the activity ratio of 1:5 (0.5 and 2.5 U) were analyzed by DNS method (Fig. 5a). *Tl*Abf51 and *Tc*Xyn10A showed significant synergistic effects. For this enzyme-activity ratio, the maximum amounts of oligosaccharides (corresponding to 37.9% hydrolysis of the cornstalk) were obtained after a 24-h hydrolysis of cornstalk (Fig. 5b; Additional file 3). Xylotriose was the major oligosaccharide produced, followed by arabinose, xylobiose, and low amounts of xylose, xylotetraose, and xylopentaose. Interestingly, the enzymes dramatically increased the oligosaccharide levels to 2.8 g/L (corresponding to 56.2% hydrolysis of the corn bran) during the hydrolysis of corn bran (Fig. 5c; Additional file 4). Among the released sugars, the most abundant were arabinose, xylose, xylobiose, and xylotriose, which differed from the hydrolysis products of cornstalk. After 36-h hydrolysis, 1.65 g/L arabinose was obtained from corn bran, which was ~ 2.8-fold more than that obtained from cornstalk.

### Effect of *Tl*Abf51 on arabinose release from cellulosic biomass

Cornstalk and corn bran were selected as cellulosic biomass, and the effect of *Tl*Abf51 and the commercial complex enzyme ULTRAFLO XL on arabinose release was compared. As shown in Additional files 5 and 6, the effect of *Tl*Abf51 on arabinose release from cornstalk was comparable with that of ULTRAFLO XL, but significantly weaker than that of ULTRAFLO XL in corn bran. Our study is in agreement with others showing the synergistic effect of Abf and cellulose/hemicellulose. The Novozymes ULTRAFLO XL has not only Abf, but also xylanase, pentosanase, cellulase, and α-amylase activity. Probably this explains the greater efficiency of the Novozymes ULTRAFLO XL observed in our study.

## Discussion

In the past decade, improvements in the utilization of renewable energy resources have attracted great interest worldwide [21]. The use of waste by-products of agricultural cereals as important sources of biomass helps avoid competition between energy and food crops [1]. For example, the by-product stream from wheat contains ~ 66% arabinoxylan by weight, of which ~ 74–91% by weight is water-soluble [22]. To increase the utilization of arabinoxylan in the waste by-products of agricultural cereals, it is important to efficiently degrade the abundant arabinoxylan into its monosaccharides, arabinose and xylose. In this study, a GH51 Abf *Tl*Abf51 was identified from *T. leycettanus* JCM12802, and its high expression was achieved in *P. pastoris* GS115. As shown in Table 1, the optimal pH (3.5–4.0) for this enzyme was similar to that for Abf from *A. kawachii* [10], but lower than those for Abfs from *C. lucknowense* C1 (5.0; [13]), *P. chrysogenum* 31B (5.0; [14]) and *Penicillium purpurogenum* (5.0; [23]). *Tl*Abf51 exhibited good stability over a wider pH range (3.0–9.0) than the other fungal GH51 Abfs. Examination of the optimal temperature and thermostability showed that *Tl*Abf51 exceeded the general properties of formerly reported counterparts that were more active at 40–60 °C and stable at 50 °C. However, compared to the other characterized fungal Abfs, *Tl*Abf51 exhibited significantly greater catalytic efficiency (*k*_cat_/*K*_m_ value; approximately 125-fold higher) towards 4-nitrophenyl-α-l-arabinofuranoside. These characteristics make *Tl*Abf51 one of the most feasible candidates for industrial applications.

**Table 1:** Property comparison of *Tl*Abf51 with other fungal Abfs of GH51

Microorganism	MW (kDa)	pH optimum	Temperature optimum (°C)	pH stability	Thermostability	*k*_cat_/*K*_m_ (/mM/s)^a^	References
*T. leycettanus* JCM12802	67.2	3.5–4.0	55–60	3.0–9.0	> 90% activity at 50 °C for 30 min	5712	This study
*Aspergillus kawachii*	80 for AkabfA 62 for AkabfB	4.0	55	3.0–7.0	Stable at 55 °C	ND	[10]
*Chrysosporium lucknowense* C1	71	5.0	40	ND	ND	ND	[13]
*Penicillium chrysogenum* 31B	71 for AFQ1 52 for AFS1	5.0	50	4.0–8.0 for AFQ1 3.0–7.0 for AFS1	> 80% activity at 50 °C for 1 h Completely inactivated at 60 °C	16.7 for AFQ1 44.9 for AFS1	[14]
*Penicillium purpurogenum*	70	5.0	60	ND	~ 50% activity at 50 °C for 2 h	24.8	[23]

*ND* not determined

^a^Catalytic efficiency (*k*_cat_/*K*_m_ value) was determined using 4-nitrophenyl-α-l-arabinofuranoside as the substrate

In terms of its substrate specificity, *Tl*Abf51 was highly specific for α-l-arabinofuranosyl linkages, as observed for homologous enzymes. *Tl*Abf51 produced arabinose as the sole hydrolysis product towards sugar beet arabinan and debranched sugar beet arabinan, but the amount liberated from the former was significantly higher than from the latter (480.7 vs. 64.4 mg/L). This suggests that *Tl*Abf51 is a type B Abf, given that type B Abfs are more active against polymeric arabinoxylan, which is singly or doubly substituted with 1,2- or 1,3-linked arabinose residues [24]. Two recent reports described bacterial GH51 Abfs isolated from *Alicyclobacillus* sp. A4 (AcAbf51A; [25]) and *Paenibacillus* sp. THS1 (THSAbf; [26]) that displayed both Abf and xylanase activities. However, 12-h digestion of water-soluble wheat arabinoxylan with *Tl*Abf51 generated arabinose as the sole product (Fig. 3a), and no xylooligosaccharides were produced. To date, no studies have demonstrated that fungal GH51 Abfs exhibit xylanase activity. The deduced amino acid sequence of *Tl*Abf51 shares 23.5% similarity with those of AcAbf51A and THSAbf (Additional file 7). Bouraoui et al. [25] demonstrated the functional importance of the catalytic dyad (Glu177 and Glu296) and hydrophobic residue Trp101 located on the β2α2 loop in THSAbf; the corresponding residues in *Tl*Abf51 are Gln199, Pro354, and Gly120, respectively. These results indicate that the function of fungal Abf is distinct from that of bacterial Abf.

To improve the hydrolysis efficiency for xylan, combinations of *Tl*Abf51 and bifunctional xylanase/cellulase *Tc*Xyn10A were examined both sequentially and simultaneously. Significant synergy occurred in all sequential reactions performed with different enzyme-activity ratios, with the highest arabinose and xylooligosaccharides production observed when *Tl*Abf51 was added after *Tc*Xyn10A or when both enzymes were added simultaneously (Fig. 3). Similar observations have been reported for AcAbf51A [24]. These results indicate that efficient degradation of wheat arabinoxylan occurred by first interrupting the main chains with *Tc*Xyn10A, followed by branch separation with *Tl*Abf51. The underlying mechanism may involve cleavage of the main chains by *Tc*Xyn10A which can make arabino-branched xylooligosaccharides more accessible to *Tl*Abf51, while removal of the side chains of arabino-branched xylooligosaccharides by *Tl*Abf51 can also improve the degradation efficiency of arabino-branched xylooligosaccharides by *Tc*Xyn10A [27]. Based on this, we tested nine enzyme-activity ratios with *Tc*Xyn10A and *Tl*Abf51 and compared their performances in wheat arabinoxylan degradation. The combination of *Tc*Xyn10A and *Tl*Abf51 at an enzyme-activity ratio of 1:5 was determined to be the most efficient (Fig. 4). It is well-known that it is difficult to degrade complex substrates such as wheat arabinoxylan which contains large amounts of arabinoxylan [28, 29]. Arabino-branched polysaccharides and xylooligosaccharides were efficiently debranched by *Tl*Abf51. Interestingly, similar activity ratios between core and accessory enzymes utilized for natural substrate degradation have been reported, such as those for polygalacturonase and pectin methylesterase from *Penicillium oxalicum* for pectin degradation [30], as well as endoglucanase and cellobiohydrolase from *Irpex lacteus* for cellulose degradation [31]. Therefore, the ideal enzyme-activity ratio (between core and accessory enzymes) for maximizing hydrolysis yields and minimizing enzyme usage in biomass degradation may be approximately 1:5. Using this enzyme-activity ratio, 1.9 and 2.8 g/L of oligosaccharides were obtained after 24-h hydrolysis of cornstalk and corn bran, respectively.

## Conclusions

An Abf from *T. leycettanus* JCM12802 was heterologously expressed and characterized. The acidity of *Tl*Abf51 exhibited good stability over a broad pH range (3.0–9.0), and *Tl*Abf51 exhibited significantly greater catalytic efficiency than other fungal GH51 Abfs. The enzyme preferentially removed 1,2- or 1,3-linked arabinose residues from arabinoxylan and acted synergistically with the bifunctional xylanase/cellulase *Tc*Xyn10A. Simultaneous addition of *Tc*Xyn10A and *Tl*Abf51 resulted in the highest degradation efficiency of wheat arabinoxylan at an enzyme-activity ratio of 1:5. Additionally, this enzyme cocktail exhibited efficient degradation of sodium hydroxide-pretreated cornstalk and corn bran. This study demonstrated the efficient enzymatic saccharification of lignocellulose and suggests the high potential of using *Tl*Abf51 in the field of biomass pretreatment.

## Methods

### Strains, vectors, and media

*Talaromyces leycettanus* JCM12802 (Japan Collection of Microorganisms RIKEN BioResource Center, Tsukuba, Japan) was cultured in medium containing lignocellulose as the sole carbon source at 45 °C for 3 days [32]. *Escherichia coli* strain Trans I-T1 and the pEASY-T3 vector (TransGen, Beijing, China) were employed for DNA manipulation. *P. pastoris* GS115 and the pPIC9 vector (Invitrogen, Carlsbad, CA, USA) were used for heterologous gene expression.

### Cloning *Tlabf51*

The full-length *Tlabf51* gene was identified in the genome of *T. leycettanus* strain JCM12802 (whole genome sequenced, unpublished). After growth for 3 days in induction medium, mycelia were collected to extract total RNA, which were further purified using the SV Total RNA isolation system (Promega, Madison, WI, USA). First-strand cDNA was generated with the ReverTra Ace-α-^®^ Kit (TOYOBO, Osaka, Japan) using purified total RNA as a template. Subsequently, the full-length cDNA of *Tlabf51* was amplified by high-fidelity PCR using specific primers (no signal peptide coding sequence based on SignalP 4.0 prediction. F: 5′-ATGAAAACCCTCCCCGCATTGGCCGGCGGC-3′; and R: 5′-CTAAGACACGGCCAGCACCGCAACAGCCCA-3′). Next, the specific gene fragment was cloned into the pEasy-T3 vector for sequencing.

### Heterologous expression and purification

The cDNA fragment encoding *Tlabf51* was amplified from the pEasy-T3-*Tlabf51* plasmid using primers with flanking restriction sites (pF: 5′-TTGAATTCATGAAAACCCTCCCCGCATTGG-3′; and pR: 5′-TAGCGGCCGCCTAAGACACGGCCAGCACCG-3′; *Eco*RI and *Not*I sites underlined, respectively). The PCR product was gel-purified, digested with corresponding restriction endonucleases, and then linked into the vector pPIC9. After verification by DNA sequencing, *Bgl*II was used to linearize the recombinant plasmid followed by electroporation to transform *P. pastoris* GS115 competent cells. Based on enzymatic activities in shake tubes, the positive transformants were screened, and the transformant with the highest Abf activity was selected for fermentation following as described by Yang et al. [27].

To remove cell debris and undissolved materials, the induced cultures were collected and centrifuged at 12,000×*g* for 10 min at 4 °C. The cell-free culture supernatant was concentrated with a 10-kDa molecular weight cutoff Vivaflow 200 membrane (Vivascience, Hannover, Germany), followed by desalination in 20 mM McIlvaine buffer (pH 3.0) using a 5-mL HiPrep desalting column. Next, the desalted sample was loaded onto a HiTrap SP HP 5-mL FPLC column (GE Healthcare), which had been pre-equilibrated with McIlvaine buffer. To obtain the target proteins, a linear gradient of NaCl (0–1.0 M) in the same buffer was used. Fractions showing enzyme activities were eluted and subjected to SDS-PAGE. The protein concentration was measured by the Bradford assay via determining the absorbance at 595 nm. Bovine serum albumin was used as the standard.

### Enzyme assay

Abf activity was determined according to the method of Yang et al. [27], with some modifications. Briefly, standard reactions contained 250 µL of 1 mM 4-nitrophenyl-α-l-arabinofuranoside and 250 μL properly diluted enzyme solution in 0.1 M McIlvaine buffer (pH 3.5). After incubation at 50 °C for 10 min, 1.5 mL 1 M Na_2_CO_3_ was added to terminate the reaction. The absorption at 405 nm was determined to detect the amount of *p*-nitrophenol released. All reactions were performed in triplicate. One unit of Abf activity was defined as the amount of enzyme that released 1 μmol of 4-nitrophenyl/min under standard conditions.

Xylanase activity was measured using the DNS method [33] with d-xylose as the standard. The reaction system containing 900 μL 1% (w/v) water-soluble wheat arabinoxylan (Megazyme) in 0.1 M McIlvaine buffer (pH 3.5) and 100 μL of an appropriately diluted enzyme solution was incubated at 55 °C for 10 min followed by the addition of 1.5 mL DNS reagent, and then the concentration of reducing sugars was determined by measuring the absorption at 540 nm. Each reaction was performed in triplicate. One unit of xylanase activity was defined as the amount of enzyme that released reducing sugars equivalent to 1 μmol of d-xylose/min under the assay conditions.

### Biochemical characterization

The pH optima in terms of the activity of purified recombinant *Tl*Abf51 was measured in 10-min reactions performed at 55 °C in 0.1 M McIlvaine buffer over a pH range of 2.5–7.0. To estimate enzyme stability at different pH levels (0.1 M McIlvaine buffer, pH 2.5–7.0; 0.1 M Tris–HCl, pH 8.0–9.0), residual activities were measured under standard conditions after the enzyme was pre-incubated in buffer without substrate at 37 °C for 1 h. To determine the optimum reaction temperature, 10-min reactions were performed at different temperatures ranging from 30 to 70 °C at pH 3.5. The thermal stability of *Tl*Abf51 was investigated by measuring residual activities under standard conditions after pre-incubation of the enzyme for 30 min at the same temperatures (as described above) in the absence of substrate.

### Substrate specificity and kinetic parameters

The substrate activities of *Tl*Abf51 on 4-nitrophenyl-glycoside substrates (Sigma; including 4-nitrophenyl-α-l-arabinofuranoside, 4-nitrophenyl-β-d-xylopyranoside, 4-nitrophenyl-α-d-galactopyranoside, 2-nitrophenyl-β-d-galactopyranoside, 4-nitrophenyl-α-d-glucopyranoside, 4-nitrophenyl-α-l-arabinopyranoside, and p-nitrophenyl-acetate) were measured by determining the Abf activity under the standard conditions described above. The substrate activities of *Tl*Abf51 on polysaccharide substrates (Megazyme; including water-soluble wheat arabinoxylan, sugar beet arabinan, and debranched sugar beet arabinan) were detected by HPAEC-PAD using a 250 × 3 mm CarboPac PA200 guard column (Thermo Fisher Scientific, Waltham, MA, USA) as previously reported [34]. Arabinose and xylooligosaccharides (xylose, xylobiose, xylotriose, xylotetraose, xylopentaose, and xylohexaose) were used as standards.

Enzyme-kinetics assays were determined at 55 °C for 5 min in 0.1 M McIlvaine buffer (pH 3.5) with 0.1–5 mM 4-nitrophenyl-α-l-arabinofuranoside as substrate. The constants (*K*_m_ and *V*_max_ values) of *Tl*Abf51 were plotted by fitting the data to a Michaelis–Menten plot using GraphPad Prism software (GraphPad, Inc., La Jolla, CA, USA).

### Synergistic hydrolysis of wheat arabinoxylan with *Tl*Abf51 and *Tc*Xyn10A

The bifunctional xylanase/cellulase *Tc*Xyn10A from *T. crustaceus* JCM12803 [20] is an excellent, economically viable candidate for the enzymatic degradation of plant cell wall polysaccharides for biofuels and bio-based chemicals. Thus, its synergistic activity with *Tl*Abf51 in the hydrolysis of wheat arabinoxylan was investigated. To study the hydrolytic activities of *Tl*Abf51 and *Tc*Xyn10A on water-soluble wheat arabinoxylan, the hydrolysis products were analyzed by HPAEC-PAD as described above. All reaction systems containing 900 μL of 0.5% (w/v) substrate and 100 μL of enzyme(s) (0.5 U each of *Tl*Abf51 and/or *Tc*Xyn10A) were performed at 37 °C in 0.1 M McIlvaine buffer (pH 4.0). After 12-h incubation, the reactions were terminated by heat denaturation by boiling for 10 min. The second enzyme solution was then added for the sequential reactions. The reaction system with substrate but without any enzyme was defined as the blank control. The resulting hydrolysis products were analyzed by the HPAEC-PAD method.

To determine the extent of synergy, different enzyme-activity ratios were used, and the production of reducing ends from water-soluble wheat arabinoxylan was measured. Experimentally, 0.5 U of *Tc*Xyn10A was combined with *Tl*Abf51 at enzyme-activity ratios ranging from 1:1 to 1:10 and incubated with 0.5% wheat arabinoxylan. The hydrolysis reactions were carried out in McIlvaine buffer at pH 4.0 and 37 °C for 12 h, and then the reactions were terminated by heat denaturation by boiling for 10 min. The amount of reducing sugars released was determined using the DNS method.

### Synergistic hydrolysis of sodium hydroxide-pretreated cornstalk and corn bran

Cornstalk and corn bran pretreatments were performed according to Zhuo et al. [5]. The milled cornstalk and corn bran samples were autoclaved at 120 °C for 1 h with 1% (w/v) sodium hydroxide at a 10% ratio (w/v). Next, pretreated samples were filtered through eight layers of gauze, and then washed multiple times with distilled water, followed by drying in a thermotank at 40 °C to achieve a constant weight for subsequent saccharification experiments. Synergistic hydrolysis of sodium hydroxide-pretreated cornstalk and corn bran was studied in 0.1 M McIlvaine buffer (pH 4.0) containing pretreated samples (0.5%, w/v), 0.5 U of *Tc*Xyn10A, and 2.5 U of *Tl*Abf51. The reaction system with substrate but without any enzyme was defined as the blank control. Hydrolysis proceeded for various durations, and the samples were collected and analyzed by HPAEC-PAD.

### Comparison of *Tl*Abf51 with a commercial enzyme

The effect of *Tl*Abf51 on arabinose release from cornstalk and corn bran was compared with that of a commercial multi-active β-glucanase from Novozymes (ULTRAFLO XL). Firstly, the Abf activity of *Tl*Abf51 and ULTRAFLO XL was evaluated under the same conditions (pH 4.0 and 55 °C). Then, mixtures of cellulosic biomass sample (cornstalk or corn bran; 0.5%, w/v) and 2.5 U enzyme (*Tl*Abf51 or ULTRAFLO XL) in 0.1 M McIlvaine buffer (pH 4.0) were incubated at 37 °C for 12 h. The released arabinose was assessed by HPAEC-PAD.

## Additional files

**Additional file 1.** SDS-PAGE analysis of the purified recombinant *Tl*Abf51. Lanes: M, the standard protein molecular weight markers; 1, the purified recombinant *Tl*Abf51.

**Additional file 2.** . LC-ESI-MS/MS analysis of the purified recombinant *Tl*Abf51.

**Additional file 3.** Time course of hydrolysis of sodium hydroxide pretreated cornstalk by simultaneously addition of *Tc*Xyn10A and *Tl*Abf51 at activity ratio of 1:5 (0.5 U and 2.5 U). 1, the oligosaccharides standards; 2–7, the hydrolysate with enzyme treatment for 3 h, 6 h, 9 h, 12 h, 24 h and 36 h, respectively.

**Additional file 4.** Time course of hydrolysis of sodium hydroxide pretreated corn bran by simultaneously addition of *Tc*Xyn10A and *Tl*Abf51 at activity ratio of 1:5 (0.5 U and 2.5 U). 1, the oligosaccharides standards; 2–7, the hydrolysate with enzyme treatment for 3 h, 6 h, 9 h, 12 h, 24 h and 36 h, respectively.

**Additional file 5.** The effect of *Tl*Abf51 on arabinose release from cornstalk was compared with that of a commercial multi-active β-glucanase from Novozymes (ULTRAFLO XL).

**Additional file 6.** The effect of *Tl*Abf51 on arabinose release from corn bran was compared with that of a commercial multi-active β-glucanase from Novozymes (ULTRAFLO XL).

**Additional file 7.** Amino acid sequence alignment of *Tl*Abf51 from *Talaromyces leycettanus* JCM12802 with other two GH51 Abfs from *Alicyclobacillus* sp. A4 (AcAbf51A) and *Paenibacillus* sp. THS1 (THSAbf).

## Data Availability

All data generated or analyzed during this study are included in this published article and its Additional files.

## Abbreviations

DP: degrees of polymerization
Abf: α-l-arabinofuranosidase
GH: glycoside hydrolase
SDS-PAGE: sodium dodecyl sulfate-polyacrylamide gel electrophoresis
LC-ESI-MS: liquid chromatography–electrospray ionization tandem mass spectrometry
*K*_m_: Michaelis constant
*V*_max_: maximum reaction rate
*k*_cat_: turnover number
HPAEC-PAD: high-performance anion-exchange chromatography and pulsed amperometric detection
