# Supplementary material for: A GH51 α-l-arabinofuranosidase from Talaromyces leycettanus strain JCM12802 that selectively drives synergistic lignocellulose hydrolysis
Source: Microb Cell Fact. 2019 Aug 19;18:138. doi: 10.1186/s12934-019-1192-z (PMC6699109; doi:10.1186/s12934-019-1192-z)
Supplement: Supplementary file 1 — Additional file 1. SDS-PAGE analysis of the purified recombinant TlAbf51. Lanes: M, the standard protein molecular weight markers; 1, the purified recombinant TlAbf51. [file 12934_2019_1192_MOESM1_ESM.docx]

**Additional Materials**

**A GH51 α-l-arabinofuranosidase from *Talaromyces leycettanus* strain JCM12802 that selectively drives synergistic lignocellulose hydrolysis**

Tao Tu*, Xiaoli Li, Kun Meng, Yingguo Bai, Yuan Wang, Zhenxing Wang, Bin Yao, Huiying Luo*

Key Laboratory for Feed Biotechnology of the Ministry of Agriculture, Feed Research Institute, Chinese Academy of Agricultural Sciences, Beijing 100081, China

^*^ Corresponding authors. Key Laboratory for Feed Biotechnology of the Ministry of Agriculture, Feed Research Institute, Chinese Academy of Agricultural Sciences, No. 12 Zhongguancun South Street, Beijing 100081, P. R. China. Tel.: +86 10 82106053; fax: +86 10 82106054.

*E-mail addresses*: [tutao@caas.cn](mailto:tutao@caas.cn); luohuiying@caas.cn

**Additional file 1.** SDS-PAGE analysis of the purified recombinant *Tl*Abf51. Lanes: M, the standard protein molecular weight markers; 1, the purified recombinant *Tl*Abf51.

**
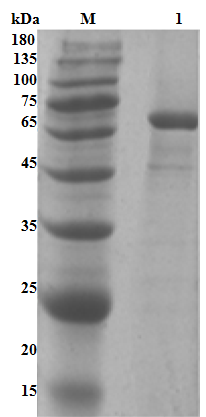
**
