# Supplementary figures and images for: A GH51 α-l-arabinofuranosidase from Talaromyces leycettanus strain JCM12802 that selectively drives synergistic lignocellulose hydrolysis
Source: Microb Cell Fact. 2019 Aug 19;18:138. doi: 10.1186/s12934-019-1192-z (PMC6699109; doi:10.1186/s12934-019-1192-z)

**Additional file 2.** LC-ESI-MS/MS analysis of the purified recombinant *Tl*Abf51.


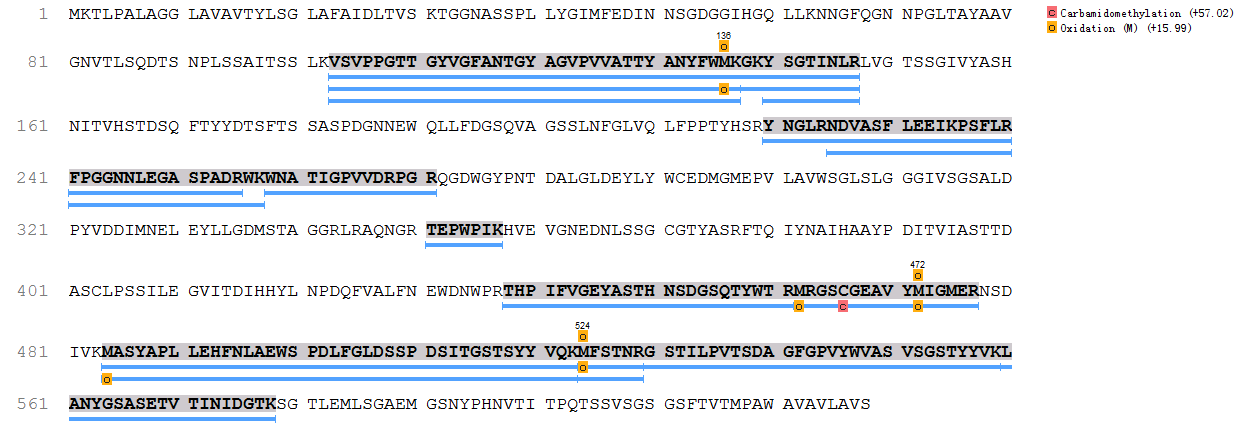

Supplement: Supplementary file 2 — Additional file 2. . LC-ESI-MS/MS analysis of the purified recombinant TlAbf51. [file 12934_2019_1192_MOESM2_ESM.docx]
