# Supplementary material for: A GH51 α-l-arabinofuranosidase from Talaromyces leycettanus strain JCM12802 that selectively drives synergistic lignocellulose hydrolysis
Source: Microb Cell Fact. 2019 Aug 19;18:138. doi: 10.1186/s12934-019-1192-z (PMC6699109; doi:10.1186/s12934-019-1192-z)

**Additional file 4.** Time course of hydrolysis of sodium hydroxide pretreated corn bran by simultaneously addition of *Tc*Xyn10A and *Tl*Abf51 at activity ratio of 1:5 (0.5 U and 2.5 U). 1, the oligosaccharides standards; 2–7, the hydrolysate with enzyme treatment for 3 h, 6 h, 9 h, 12 h, 24 hand 36 h, respectively.


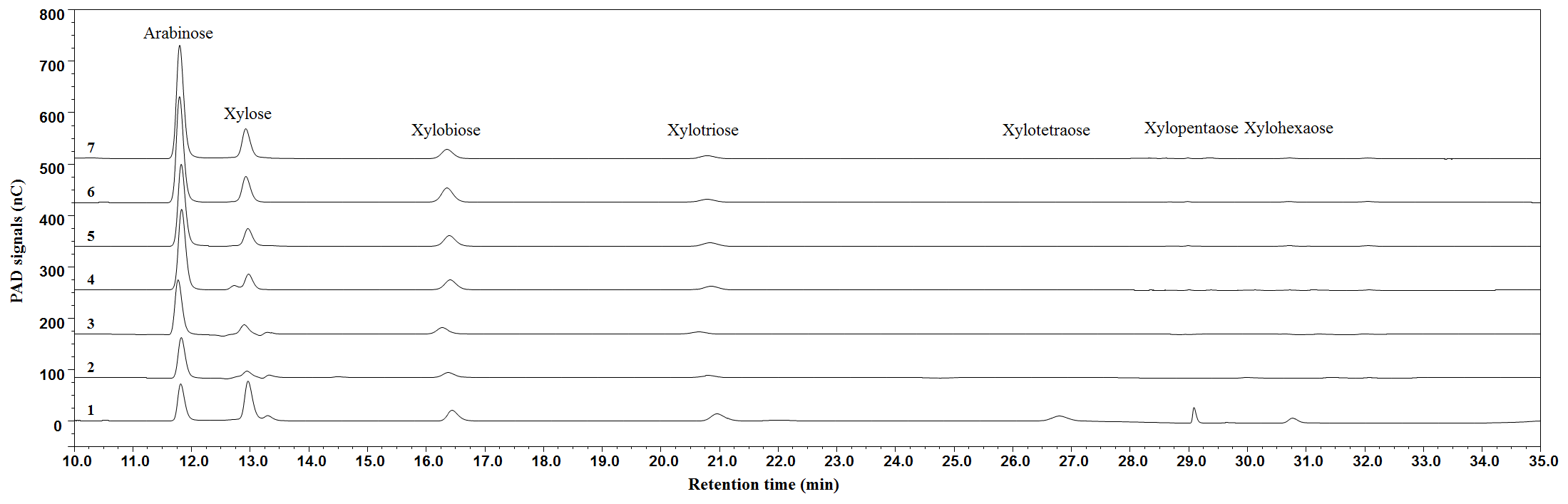

Supplement: Supplementary file 4 — Additional file 4. Time course of hydrolysis of sodium hydroxide pretreated corn bran by simultaneously addition of TcXyn10A and TlAbf51 at activity ratio of 1:5 (0.5 U and 2.5 U). 1, the oligosaccharides standards; 2–7, the hydrolysate with enzyme treatment for 3 h, 6 h, 9 h, 12 h, 24 h and 36 h, respectively. [file 12934_2019_1192_MOESM4_ESM.docx]
