# Supplementary material for: A GH51 α-l-arabinofuranosidase from Talaromyces leycettanus strain JCM12802 that selectively drives synergistic lignocellulose hydrolysis
Source: Microb Cell Fact. 2019 Aug 19;18:138. doi: 10.1186/s12934-019-1192-z (PMC6699109; doi:10.1186/s12934-019-1192-z)

**Additional file 5.** The effect of *Tl*Abf51 on arabinose release from cornstalk was compared with that of a commercial multi-active β-glucanase from Novozymes (ULTRAFLO XL).


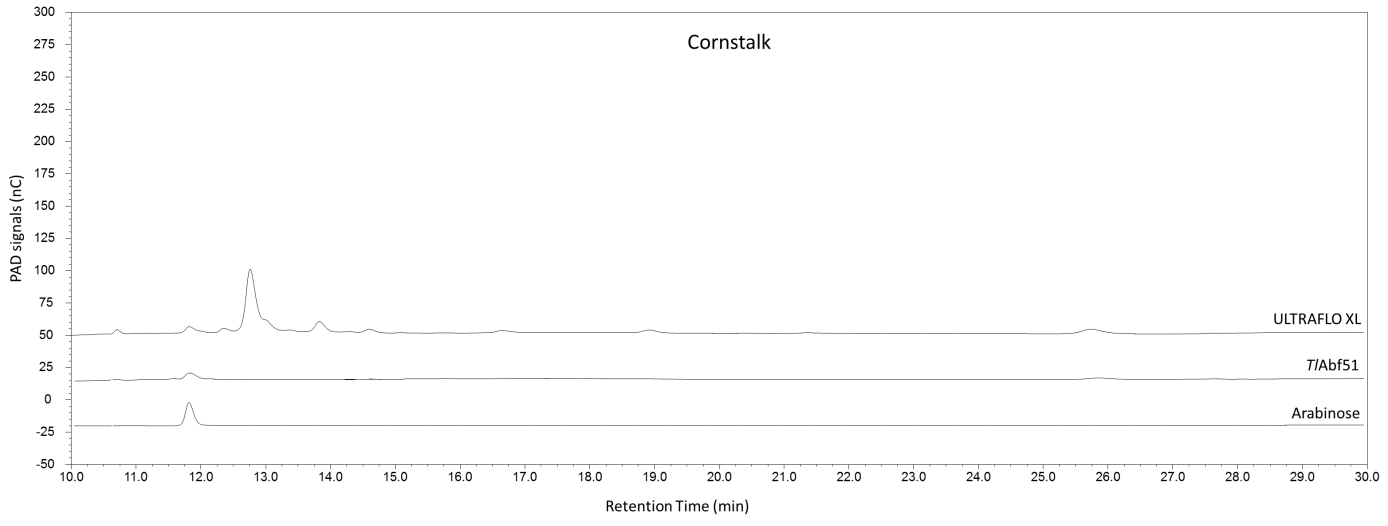

Supplement: Supplementary file 5 — Additional file 5. The effect of TlAbf51 on arabinose release from cornstalk was compared with that of a commercial multi-active β-glucanase from Novozymes (ULTRAFLO XL). [file 12934_2019_1192_MOESM5_ESM.docx]
