# Supplementary material for: A GH51 α-l-arabinofuranosidase from Talaromyces leycettanus strain JCM12802 that selectively drives synergistic lignocellulose hydrolysis
Source: Microb Cell Fact. 2019 Aug 19;18:138. doi: 10.1186/s12934-019-1192-z (PMC6699109; doi:10.1186/s12934-019-1192-z)

**Additional file 7.** Amino acid sequence alignment of *Tl*Abf51 from *Talaromyces leycettanus* JCM12802 with other two GH51 Abfs from *Alicyclobacillus* sp. A4 (AcAbf51A) and *Paenibacillus* sp. THS1 (THSAbf).


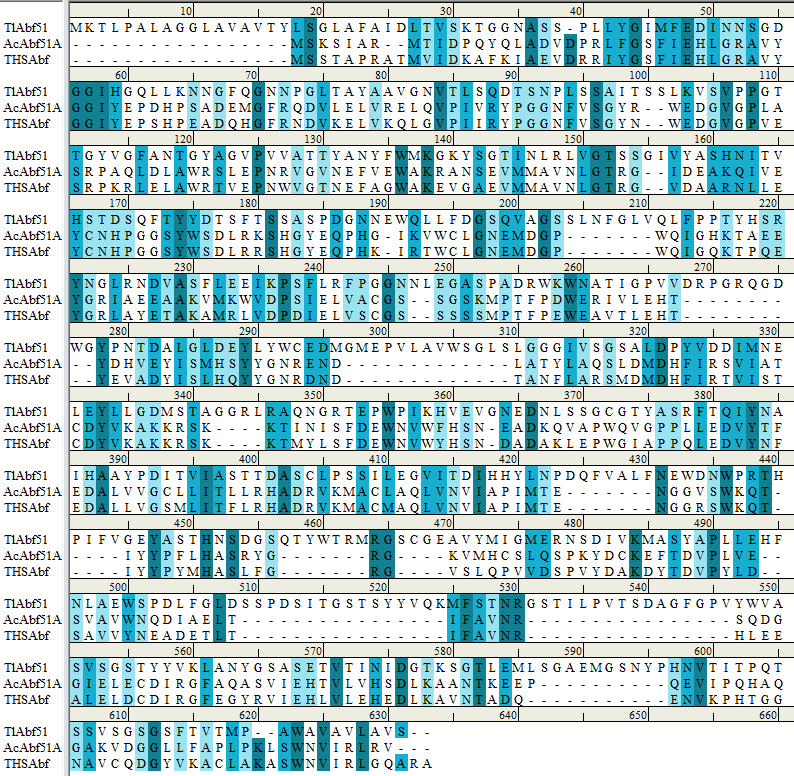

Supplement: Supplementary file 7 — Additional file 7. Amino acid sequence alignment of TlAbf51 from Talaromyces leycettanus JCM12802 with other two GH51 Abfs from Alicyclobacillus sp. A4 (AcAbf51A) and Paenibacillus sp. THS1 (THSAbf). [file 12934_2019_1192_MOESM7_ESM.docx]
